# Eating disorders and emotional dysregulation are associated with insufficient weight loss after bariatric surgery: a 1-year observational follow-up study

**DOI:** 10.1007/s40519-023-01574-z

**Published:** 2023-06-02

**Authors:** Margherita Barbuti, Giulia Carignani, Francesco Weiss, Alba Calderone, Paola Fierabracci, Guido Salvetti, Giulia Menculini, Alfonso Tortorella, Ferruccio Santini, Giulio Perugi

**Affiliations:** 1 2nd Psychiatry Unit, Department of Clinical and Experimental Medicine, University Hospital of Pisa, Via Savi 10, 56126 Pisa, Italy; 2 1st Endocrinology Unit, Department of Clinical and Experimental Medicine, Obesity and Lipodystrophy Research Center, University Hospital of Pisa, Via Paradisa, 2, 56124 Pisa, Italy; 3 Section of Psychiatry, Department of Medicine and Surgery, University of Perugia, Piazza Lucio Severi 1, 06132 Perugia, Italy

**Keywords:** Obesity, Bariatric surgery, Weight loss, Mood disorders, Binge eating disorder, Emotional dysregulation

## Abstract

**Purpose:**

Subjects with obesity, especially those seeking bariatric surgery, exhibit high rates of mental disorders and marked psychopathological traits. The primary objective of this prospective, non-interventional study was to investigate whether the presence of different psychiatric disorders, attention deficit/hyperactivity disorder (ADHD) symptomatology and emotional dysregulation influenced weight loss at 1-year follow-up after surgery.

**Methods:**

Subjects consecutively referred for pre-surgical evaluation at the Obesity Center of Pisa University Hospital were recruited. Psychiatric diagnoses were made through the Mini-International Neuropsychiatric Interview (MINI) and ADHD symptomatology was assessed with the Wender–Reimherr Adult Attention Deficit Disorder Scale (WRAADDS). Emotional dysregulation was investigated through the WRAADDS and self-report questionnaires. After surgery, weight and obesity-related comorbidities were monitored during follow-up.

**Results:**

Of the 99 participants recruited, 76 underwent surgery and 65 could be reevaluated 1 year after surgery. Subjects with insufficient weight loss (excess body mass index loss ≤ 53%, *n* = 15) had more frequent lifetime binge eating disorder (BED) and BED-mood disorders comorbidity than subjects with favorable post-surgical outcome. Additionally, they scored higher on both physician-administered and self-report scales assessing emotional dysregulation, which represents a nuclear symptom of ADHD in adults. At the logistic regression analysis, older age, higher preoperative excess body mass index and greater affective instability were predictors of reduced weight loss at 1-year follow-up.

**Conclusion:**

Emotional dysregulation seems to be associated with a worse outcome after bariatric surgery. Further studies with larger samples and longer follow-up are needed to confirm the influence of different psychiatric disorders and psychopathological traits on post-surgical outcome.

**Level of evidence:**

V, prospective descriptive study.

## Introduction

Bariatric surgery is currently the most effective and long-lasting treatment for severe and complex obesity and is associated with a significant improvement in weight-related comorbidities and reduced mortality rates [1]. However, post-surgery weight outcome varies widely, even among subjects undergoing the same procedure. A variable percentage of subjects (20–25%) experience insufficient weight loss 1 year after surgery, often defined as an excess BMI loss ≤ 50% of the initial excess BMI [2]. The reasons for this wide variability in response to bariatric procedures remain largely unpredictable on an individual level. Indeed, failure to achieve satisfactory weight loss involves a number of surgical and non-surgical factors, including psychiatric and behavioral aspects [3].

Although no conclusive data have been produced, there is strong evidence of a close relationship between obesity and various psychiatric disorders, especially in bariatric populations [4]. Psychiatric assessment is widely recommended during the multidisciplinary evaluation performed prior to bariatric surgery [5]. In fact, psychiatric disorders are thought to have an impact on various post-surgical outcomes, including weight loss and quality of life, both in the short- and in the long-term [6].

However, only few methodologically rigorous studies utilizing structured interviews have examined the relationship between psychiatric disorders and weight loss following different types of bariatric surgery. Some of these have found postoperative poor weight loss in the presence of preoperative mood or eating disorders, in particular binge eating disorder (BED), whereas other studies found no association between these conditions and surgical outcomes [7, 8].

Furthermore, recent studies have focused on the role of some trans-nosographic psychopathological traits as predictors of weight loss program success and bariatric surgery outcomes, such as attention deficit/hyperactivity disorder (ADHD) symptoms and emotional dysregulation [9–11]. Obesity, BED, and ADHD have been hypothesized to share common underlying neurobiological and neuropsychological abnormalities such as dysfunctions in brain reward pathways, emotion regulation processes and executive functions that could affect post-surgical behavior and outcome [12]. Indeed, difficulty in emotion regulation has been found to be independently associated with emotional overeating and general eating pathology. It has been suggested that chronic negative affective states may result in the acquisition of maladaptive coping strategies and eating behaviors [13].

Consistent with these observations, recent literature on personality traits influencing bariatric surgery reports that the domain of externalizing dysfunction (e.g., poor impulse control and low frustration tolerance) is negatively associated with weight reduction at 12 months after surgery and correlates positively with maladaptive eating behavior at 3 months [14]. Borderline personality disorder, in which emotional dysregulation is a central factor, has also been considered a possible negative predictor of post-surgical weight loss [15]. On the other hand, greater impulse control ability emerged as a robust predictor of good post-surgical outcomes, regardless of psychiatric comorbidity [16].

Currently, pre-surgical psychopathological or behavioral predictors of poor post-surgical outcome have not yet been consistently demonstrated [6].

The main objective of this naturalistic study was to evaluate whether the presence of different psychiatric disorders, ADHD symptomatology and emotional dysregulation in candidates for bariatric surgery would influence the 1-year post-surgical outcome.

## Materials and methods

### Sample and study design

In this observational, prospective study, participants with obesity and seeking bariatric surgery were consecutively enrolled between March 2019 and February 2021 at the Obesity Center of the 1^st^ Endocrinology Unit in Pisa University Hospital. Patients were recruited during the psychiatric evaluation, which is routinely performed in Day Hospital setting before bariatric surgery, along with endocrinological, psychological, and nutritional examinations.

Inclusion criteria were: age ≥ 18 years, class III obesity (BMI ≥ 40 kg/m^2^) or class II obesity (BMI ≥ 35 kg/m^2^) plus medical comorbidity, and written informed consent obtained from the patient for the study participation. Patients unable to complete self-questionnaires or with unstable and/or severe medical or psychiatric conditions (e.g., acute-phase psychotic disorders) were excluded from recruitment. Other psychiatric conditions (e.g., anxiety disorders, neurodevelopmental disorders) were not considered as exclusion criteria.

Participants undergoing bariatric surgery were subsequently monitored for weight and obesity-related comorbidities during follow-up visits at 1 month (± 1 week), 3 months (± 1 week), 7 months (± 2 weeks), and 12 months (± 2 weeks) after surgery.

All subjects provided written informed consent for the study participation. This study was carried out in accordance with the Code of Ethics of the World Medical Association (Declaration of Helsinki) and the study protocol was approved by the Ethic Committee of the University (Protocol n. 23933; 29/03/2019).

### Data collection and clinical assessment

During the baseline assessment, participating psychiatrists collected sociodemographic data (age, sex, marital status, education, working status) along with several clinical variables (e.g., neurophysical development, familiarity for psychiatric disorders, eventual psychopharmacological treatment).

Current and/or lifetime psychiatric comorbidity according to the Diagnostic and Statistical Manual of Mental Disorders, 5^th^ ed. (DSM-5) criteria was assessed through the Mini-International Neuropsychiatric Interview (MINI, version 7.0.2.), a brief, structured diagnostic interview to meet the need for a short but accurate psychiatric evaluation [17].

The Wender–Reimherr Adult Attention Deficit Disorder Scale (WRAADDS), a clinician-rated scale based on the Utah Criteria for ADHD in adults, was performed to assess ADHD symptoms severity across 7 domains (Attentional difficulties, Persistent motor hyperactivity, Hot temper, Affective lability, Emotional over-reactivity, Disorganization, and Impulsivity) [18].

Emotional dysregulation was measured using the Reactivity, Intensity, Polarity, and Stability questionnaire in its 40-item version (RIPoSt-40) [19]. Items were summed to compute four subscales scores (affective instability, positive and negative emotionality, and emotional impulsivity), and a second-order negative emotional dysregulation (NED) score made up of affective instability, negative emotionality, and emotional impulsivity subscales. Finally, affective temperaments (depressive, cyclothymic, hyperthymic, irritable, anxious) were investigated through the Temperament Evaluation of Memphis, Pisa, Paris, and San Diego (Brief-TEMPS) self-questionnaire [20]. Both self-questionnaires were validated in the Italian language [19, 20]. The Cronbach's α coefficients of the RIPoSt-40 subscales for the study population are as follows: 0.900 for the affective instability subscale, 0.866 for the positive emotionality subscale, 0.837 for the negative emotionality subscale, and 0.859 the emotional impulsivity subscale. The Cronbach's α coefficients of the Brief-TEMPS subscales for the study population are as follows: 0.751 for the depressive subscale, 0.786 for the cyclothymic subscale, 0.856 for the hyperthymic subscale, 0.858 for the irritable subscale, and 0.750 for the anxious subscale.

Information concerning the BMI at the time of surgery and the type of intervention was obtained from medical records. Subsequently, during each follow-up visit, BMI was measured, and participants were interviewed about their eating habits after the intervention, any surgery-related side effects, and adherence to the dietary regimen. Any changes in drug treatment were recorded. Due to the Coronavirus disease 2019 (COVID-19) pandemic, some of the follow-up assessments were conducted via televisits.

To calculate weight loss after surgery, we used the percentage of excess BMI lost (%EBMIL). The %EBMIL is currently the best method for comparing different treatments for obesity; indeed, it most strongly correlates with improvement in metabolic syndrome after gastric bypass [21]. The ideal body weight was calculated using a BMI of 25 kg/m^2^. The %EBMIL was calculated using the formula: [%EBMIL = (initial BMI—follow-up BMI)/(initial BMI—25) × 100].

### Statistical analysis

Descriptive statistics were used to summarize sample characteristics and were reported in terms of mean and standard deviations (sd) for continuous variables with normal distribution, median and interquartile range (IQR) for nonparametric continuous variables, and number and percentages for categorical variables. We used the Shapiro–Wilk test to check the normality of continuous variables. Pairwise comparisons between baseline and 1-year assessments for mean BMI, mean excess BMI, and percentages of participants receiving pharmacological treatment were conducted by means of paired t-test for continuous variables and McNemar test for categorical ones. Next, participants with a %EBMIL below the 25th percentile (< 53%) 1 year after surgery were compared with participants who experienced adequate weight loss. Comparisons between these two independent groups were conducted using Chi-square test for categorical variables and Student’s *t*-test for continuous variables. Mann–Whitney *U* test was used to compare continuous variables that were not normally distributed. Statistical significance was settled at *p* < 0.05 (2-tailed). Given the large number of comparisons and small sample size, we added Benjamini–Hochberg correction for statistical significance in univariate comparisons. A stepwise backward logistic regression model was used to identify the predictive value of clinical characteristics on the presence of %EBMIL < 53% at the end of 1-year follow-up. A statistical significance after Benjamini–Hochberg correction in univariate comparisons was used as a threshold for inclusion of a variable in the regression model. We used the statistical routines of IBM SPSS Statistics for Mac, Version 25.0 (SPSS Inc., USA).

## Results

**Fig. 1:**
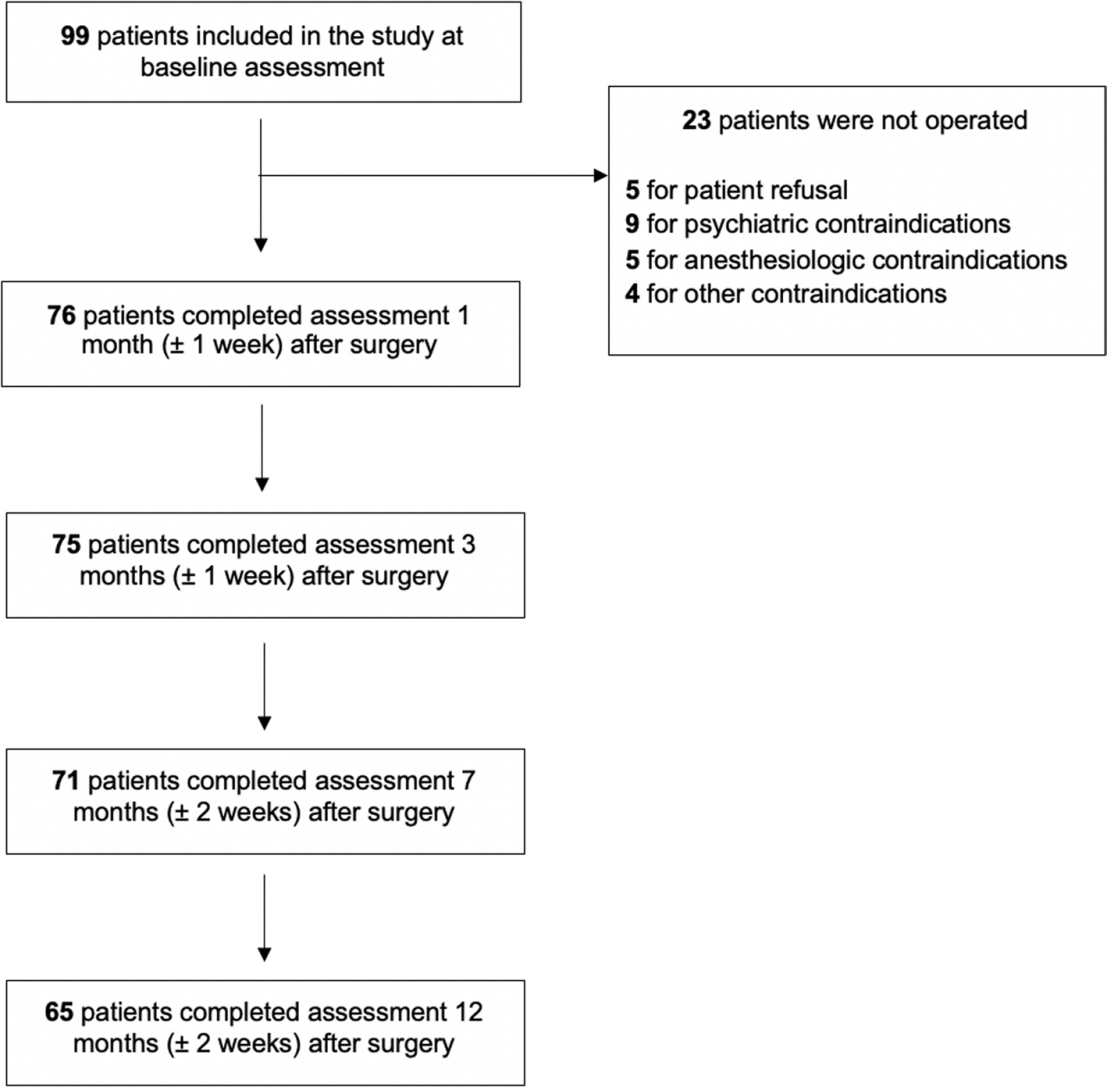
Flowchart of subjects during the 1-year follow-up after bariatric surgery

Of the 99 participants initially recruited, 76 undergo bariatric surgery while 23 were not operated for various reasons (Fig. 1). At the end of the 1-year follow-up after surgery, 65 participants could be reevaluated, whereas 11 subjects were lost to follow-up (14% of those who underwent the intervention). We found no significant differences between the individuals lost to follow-up and those who remained in the study until the final evaluation in terms of age, sex, BMI at time of surgery, and psychiatric comorbidities, although a trend of higher rate of male sex and psychiatric comorbidity in subjects lost to follow-up was noted. The present study focuses on the 65 participants who completed the 1-year follow-up.

### Baseline assessment

Female participants accounted for more than three-quarters of the sample (78.5%). The mean age was approximately 45 years (range 21–64), with a normal distribution and no significant difference between sex groups. The mean BMI was 44.9 kg/m^2^ (range 34.6–64.7) and 74% of participants had a BMI ≥ 40 kg/m^2^ (Table 1). Lifetime maximum BMI averaged 47 kg/m^2^, with a range of 37 to 69. The mean age of reported obesity onset was 16 years (range 3–52), and 36% of participants reported a history of childhood obesity (< 10 years).

**Table 1:** Clinical characteristics of the obese bariatric sample (*n* = 65) at baseline assessment

Age (mean, sd)	45.18 (11.72)
Female gender (*n*, %)	51 (78.5%)
BMI, Kg/m^2^ (mean, sd)	44.87 (6.43)
BMI, Kg/m^2^ (range)	34.57–64.74
Maximum lifetime BMI, kg/m^2^ (mean, sd)	46.97 (7.06)
Age at obesity onset, years (mean, sd)	16.31 (9.05)
Childhood obesity (*n*, %)	23 (35.9%)
Family history of obesity (*n*, %)	48 (75.0%)
Previous weight-loss pharmacological therapy (*n*, %)	27 (41.5%)
Previous bariatric surgery (*n*, %)	8 (12.3%)
Lifetime psychiatric comorbidities (*n*, %)	
Any psychiatric disorder	51 (78.5%)
Neurodevelopmental disorder	5 (7.7%)
Any mood disorders	41 (63.1%)
Major depressive disorder	16 (24.6%)
Bipolar II disorder	25 (38.5%)
Any eating disorders	34 (52.3%)
Binge eating disorder	32 (49.2%)
Bulimia nervosa	9 (13.8%)
Any anxiety disorders	27 (41.5%)
Panic disorder	21 (32.3%)
Agoraphobia	11 (16.9%)
Social phobia	8 (12.3%)
Current pharmacological treatment (*n*, %)	
Mood stabilizing agents	10 (15.4%)
Antidepressant agents	19 (29.2%)
Benzodiazepines	7 (10.8%)
Oral hypoglycemic agents	21 (32.3%)
Lipid-lowering agents	6 (9.2%)
Antihypertensive agents	21 (32.3%)

*BMI* body mass index

As for psychiatric comorbidity, almost 80% of the total sample had at least one lifetime psychiatric disorder and nearly two-thirds of participants were diagnosed with mood disorders. Twenty-six participants (63% of those with mood disorder) presented with both lifetime mood disorder and binge eating disorder (BED). BED was the most frequent single diagnosis, occurring in half of the participants (49%). Anxiety disorders were encountered in 42% of the sample. Among these, panic disorder was the most frequently detected, followed by agoraphobia and social phobia. Comorbidity with substance use disorder was uncommon (9%), and no patients were diagnosed with obsessive–compulsive disorder or psychotic disorder. Six subjects had a history of post-partum depression (13% of females) and one participant reported previous suicide attempts. Finally, five participants (8%) reported a history of ADHD diagnosed in childhood.

Forty-three percent of participants were on psychopharmacological treatment at the time of evaluation, mainly with selective serotonin reuptake inhibitors, mood stabilizing anticonvulsants, and benzodiazepines. Only 1 subject took antipsychotics, and 1 stimulants for the treatment of ADHD.

### Follow-up analyses

All surgical procedures were performed laparoscopically and none of the subjects were converted to open surgery. Forty-two subjects received sleeve gastrectomy (64.6%) and 23 Roux-en-Y gastric bypass (35.4%). The mean BMI at time of surgery was 44.8 kg/m^2^ (range 35.1–64.7). One year after surgery, the excess BMI lost averaged 13.84 kg/m^2^ (sd, 4.82), corresponding to 30% of the initial BMI and 70% of the initial excess BMI. With regard to drug therapies for obesity-related complications, only the prescription of oral hypoglycemic agents was significantly reduced 1 year after surgery compared with baseline (1.5% vs 32.3%, *p* < 0.001). None of the participants had major surgical complications or required reoperation. The most common side effects were diarrhea/stypsis (in 60% of the subjects) vomiting/regurgitation (at least one episode in 56.9% of the subjects), gastroesophageal reflux (in 33.8% of the subjects).

Fifteen of 65 participants (23.1%) showed %EBMIL under the 25th percentile (i.e., < 53%). Subjects with insufficient weight loss (%EBMIL < 53%) showed older age and higher excess BMI at time of surgery compared to participants with optimal weight outcome. In addition, sleeve gastrectomy was significantly more often performed in participants with insufficient weight loss compared to the others. We found no difference in sex distribution and age at obesity onset between the two groups of subjects (Table 2).

**Table 2:** Comparisons of clinical variables between patients who lost < 53% and patients who lost ≥ 53% of excess BMI at 1- year after bariatric surgery (*n* = 65)

	%EBMIL ≥ 53% (*n* = 50, 76.9%)	%EBMIL < 53% (*n* = 15, 23.1%)	χ^2^/*t*	OR (95% C.I.)/Cohen’s d	*p*
Age (mean, sd)	43.85 (12.04)	53.00 (6.47)	9.046	0.95	0.004**
Female sex (*n*, %)	39 (78.0%)	12 (80.0%)	0.027	1.13 (0.27–4.72)	0.869
Excess BMI (at time of surgery) (mean, sd)	19.15 (5.34)	24.49 (6.78)	2.360	0.88	0.002**
Current sleeve gastrectomy (*n*, %)	28 (56.0%)	14 (93.3%)	7.034	0.91 (0.11–0.75)	0.008**
Lifetime psychiatric comorbidities (*n*, %)					
Major depressive disorder	10 (20.0%)	6 (40.0%)	2.487	2.67 (0.77–9.25)	0.115
Bipolar disorder	19 (38.0%)	6 (40.0%)	0.020	1.09 (0.33–3.54)	0.889
Binge eating disorder	21 (42.0%)	11 (73.3%)	4.532	3.80 (1.06–13.59)	0.033*
Bulimia nervosa	6 (12.0%)	3 (20.0%)	0.619	1.83 (0.40–8.43)	0.431
Mood disorders and binge eating disorder	16 (32.0%)	10 (66.7%)	5.778	4.25 (1.25–14.50)	0.016**
Anxiety disorders	20 (40.0%)	7 (46.7%)	0.211	1.31 (0.41–4.19)	0.646
Any psychiatric disorder	38 (76.0%)	13 (86.7%)	0.777	2.05 (0.40–10.41)	0.378

*BMI* body mass index, *EBMIL* excess BMI loss

*p < 0.05; **significant after Benjamini–Hochberg correction

Regarding lifetime psychiatric comorbidities, insufficient weight loss was associated with BED (although the comparison lost statistical significance after Benjamini–Hochberg correction) and comorbidity of BED and mood disorders (i.e., major depressive disorder or bipolar disorder). Conversely, anxiety disorders or any psychiatric disorders did not show a relationship with weight loss outcome. Furthermore, no difference was found between the two groups regarding the psychopharmacological therapies prescribed during the follow-up.

Participants with reduced weight loss at 1-year follow-up did not present a higher burden of overall ADHD symptomatology than those who showed a more favorable weight outcome, as assessed by the WRAADDS performed by the participating psychiatrists. However, among the symptom domains of ADHD, affective lability and emotional over-reactivity were more frequent in subjects with reduced weight loss at 1-year follow-up compared to the others (Table 3), although these associations did not reach statistical significance after Benjamini–Hochberg correction.

**Table 3:** Comparison of ADHD symptoms, assessed by WRAADDS clinical interview, between patients who lost < 53% and patients who lost ≥ 53% of excess BMI at 1 year after bariatric surgery (*n* = 65)

	%EBMIL ≥ 53% (*n* = 50, 76.9%)	%EBMIL < 53% (*n* = 15, 23.1%)	*Z/t*	Cohen’s d	*p*
ADHD symptoms: WRAADDS					
Attentional difficulties (median, IQR)	2.00 (2.00)	2.00 (3.00)	0.573	0.16	0.567
Persistent motor hyperactivity (median, IQR)	0.00 (2.00)	1.00 (2.00)	0.039	0.01	0.969
Hot temper, explosive short-lived outbursts (median, IQR)	3.00 (3.00)	1.00 (4.00)	1.477	0.43	0.140
Affective lability (median, IQR)	2.00 (3.00)	4.00 (2.00)	2.426	0.73	0.015*
Emotional over-reactivity (median, IQR)	3.00 (3.00)	4.00 (1.00)	2.429	0.73	0.016*
Disorganization, inability to complete tasks (median, IQR)	2.00 (3.00)	2.00 (3.00)	1.514	0.438	0.130
Impulsivity (median, IQR)	3.00 (2.00)	3.00 (2.00)	1.202	0.34	0.229
WRAADDS total score (mean, sd)	14.02 (6.10)	17.33 (6.44)	1.865	0.53	0.062

*ADHD* attention deficit/hyperactivity disorder, *BMI* body mass index, *EBMIL* excess BMI loss, *WRAADDS* Wender–Reimherr Adult Attention Deficit Disorder Scale

*p < 0.05

In addition, subjects with insufficient weight loss showed a higher burden of affective instability, negative emotionality and overall emotional dysregulation on the self-questionnaires completed at baseline assessment (Table 4). Finally, a worse post-surgical outcome was associated with the presence of a markedly cyclothymic temperament.

**Table 4:** Comparison of affective temperaments and emotional dysregulation, assessed by self-questionnaires, between patients who lost < 53% and patients who lost ≥ 53% of excess BMI at 1 year after bariatric surgery (*n* = 65)

	%EBMIL ≥ 53% (*n* = 50, 76.9%)	%EBMIL < 53% (*n* = 15, 23.1%)	*t*	Cohen’s d	*p*
Emotional dysregulation: RIPoSt-40 (mean, sd)					
Affective instability	23.25 (8.00)	30.17 (10.00)	2.515	0.76	0.015**
Positive emotionality	38.31 (10.12)	42.33 (6.46)	1.299	0.47	0.199
Negative emotionality	23.57 (7.01)	29.75 (6.54)	2.743	0.91	0.008*
Emotional impulsivity	16.84 (6.20)	19.67 (4.35)	1.478	0.53	0.145
RIPoSt NED score	63.66 (17.74)	79.58 (18.13)	2.743	0.89	0.008**
RIPoSt-40 total score	101.98 (23.89)	121.92 (20.01)	2.645	0.90	0.011**
Affective temperaments: Brief-TEMPS (mean, sd)					
Depressive	14.36 (4.44)	14.83 (4.55)	0.323	0.10	0.748
Cyclothymic	10.79 (4.17)	14.75 (6.03)	2.632	0.76	0.011**
Hyperthymic	21.70 (6.27)	21.92 (3.68)	0.112	0.04	0.912
Irritable	11.34 (4.25)	11.42 (3.69)	0.056	0.02	0.955
Anxious	13.18 (4.06)	15.50 (6.84)	1.495	0.41	0.141

*BMI* body mass index, *Brief-TEMPS* Brief temperament evaluation of Memphis, *Pisa* Paris and San Diego, *EBMIL* excess BMI loss, *NED* negative emotion dysregulation, *RIPoSt-40* reactivity, intensity, polarity and stability questionnaire

*p < 0.05; **significant after Benjamini–Hochberg correction

In the multivariate logistic regression analysis, we included the following variables: age, excess BMI at the time of surgery, type of surgery (sleeve gastrectomy or gastric bypass), BED-mood disorders comorbidity, cyclothymic temperament score, RIPoSt-40 affective instability score, RIPoSt-40 NED score and RIPoSt-40 total score. The clinical features that significantly differentiated subjects with %EBMIL < 53% from subjects with adequate weight-loss, at the 1-year evaluation, were age [OR (95%C.I.) = 1.17 (1.05–1.31); Wald = 7.619; *p* = 0.006], excess BMI [OR (95%C.I.) = 1.22 (1.04–1.42); Wald = 6.394; *p* = 0.011], and RIPoSt-40 affective instability score [OR (95%C.I.) = 1.14 (1.03–1.27); Wald = 6.454; *p* = 0.011].

## Discussion

Consistent with previous literature [22], bariatric surgery has shown to be an effective treatment for morbid obesity and type II diabetes. Indeed, only less than a quarter of subjects presented with insufficient weight loss at 1-year follow-up, and oral hypoglycemic therapy was discontinued in almost all subjects. Finally, no major complications or deaths were recorded during follow-up, confirming the low risk of laparoscopic bariatric procedures [23].

To date, there is still conflicting evidence regarding the impact of certain variables on post-surgical outcome [24]. In agreement with our findings, advanced age has often been reported as a predictor of reduced weight loss after surgery [2, 25, 26]. However, this unfavorable effect of age, coupled with an increased risk of complications, does not appear to be robust enough to completely exclude older individuals from bariatric surgery [27]. Excess preoperative BMI is also considered one of the most robust negative predictors of poor weight outcome [28–30]. In fact, subjects with a higher initial BMI end up having a higher BMI after the intervention despite losing more kilograms than those with a lower initial BMI [31]. In addition, in the past, women were found to experience greater post-surgical weight loss compared with men, whereas recent results regarding the predictive value of sex, including ours, disprove this finding [6]. Finally, sleeve gastrectomy has often been associated with less weight loss than gastric bypass, although a recent meta-analysis reported similar results for both procedures [32, 33]. However, subjects undergoing laparoscopic sleeve gastrectomy have fewer postoperative complications than those undergoing laparoscopic gastric bypass. In contrast, the latter type of operation appears to be associated with greater remission of obesity-related comorbidities such as dyslipidemia, hypertension and type 2 diabetes [33, 34]. Therefore, the decision to perform bariatric surgery and the choice of procedure type must consider a number of clinical factors.

In line with previous evidence [4, 35], subjects undergoing bariatric surgery showed high rates of lifetime psychiatric comorbidities according to the DSM-5 criteria. Several studies published in recent years have reported suboptimal weight loss after bariatric surgery in subjects with lifetime psychiatric disorders [6, 36–38], although results are still conflicting [8, 39, 40]. Some Authors have reported more unsatisfactory therapeutic outcomes in subjects with BED than without this eating disorder, especially in terms of weight loss and metabolic risk reduction [28, 40–43]. On the other hand, a recent systematic review found that pre-bariatric BED appears to have little or no influence on weight loss after surgery, but also pointed out that many gaps remain due to high heterogeneity among studies and the use of different measures to assess BED [44]. It could be speculated that although binge eating is physically impossible immediately after surgery without provoking vomit/regurgitation, the tendency to lose control over eating typical of BED individuals may persist, leading to the development of other maladaptive eating behaviors (grazing, snacking, night eating) and consequently poor weight outcomes.

To the best of our knowledge, this is the first study to highlight the association between BED-mood disorder comorbidity and a worse outcome one year after the intervention. Indeed, BED and mood disorders are often comorbid in clinical practice, particularly in bariatric samples [4]. It has been hypothesized that subjects with mood disorders have an initial post-surgical “honeymoon phase” where they achieve the required weight loss results and experience a period of mood improvement, which might be associated with good control over their eating habits. Subsequently, and especially in the long term, mood disorders may relapse, sometimes with more severe affective episodes, which may favor the reappearance of eating behavior disturbances [45].

Previous studies focusing on the relationship between specific diagnoses and post-operative outcomes may have failed to account for other psychopathological constructs that may be shared among individual diagnoses and which could be particularly relevant to weight loss outcome. In our sample, participants with insufficient weight loss exhibited a higher burden of emotion regulation difficulties than subjects with a successful surgical outcome and, in particular, greater affective instability.

Both mood disorders, especially of the bipolar spectrum, and BED share a substrate of affective instability, negative emotionality, impulsivity, and anxiety [7, 46]. These latter are typical characteristics of the cyclothymic-anxious-sensitive temperament that constitute the life-long, inter-episodic psychopathologic ground of many bipolar II subjects, especially among women [46]. Our finding of a more pronounced cyclothymic temperament in subjects with reduced weight loss at 1 year is in line with these assumptions.

On the other hand, weight outcome after bariatric surgery does not seem to be influenced by the symptom domains of adult ADHD syndrome, with the exception of a slight association between insufficient weight loss and emotional dysregulation domains (affective lability and emotional hyperreactivity). This association could become more significant in studies with larger numbers of patients. ADHD is the most common neurodevelopmental disorder diagnosed in childhood and persists into adolescence and adulthood in 40%-50% of cases [47]. In adults, hyperactivity usually improves, while inattention, impulsivity, and particularly emotional dysregulation persist or even worsen. Emotional dysregulation is the feature of ADHD that most predisposes to the development of psychiatric comorbidities and addictive behaviors, such as mood and eating disorders, which can affect weight loss after surgery [1, 48].

In conclusion, the presence of marked affective instability, which is one of the nuclear components of emotional dysregulation, is a predictor of worse weight outcome after bariatric surgery, along with increased age and higher preoperative excess BMI. Individuals with a history of emotional and mood dysregulation are probably vulnerable to develop maladaptive eating and to return very soon after surgery to their pathological eating behaviors, which do not necessarily include binge eating but can still compromise long-term weight control. Although the study should be considered preliminary, our observations confirm the importance of presurgical psychiatric evaluation in subjects undergoing bariatric surgery and, more importantly, emphasize the relevance of careful postsurgical follow-up in subjects presenting with affective instability, often associated with mood and eating disorders. Further studies are urgently needed to systematically investigate psychiatric disorders and psychopathological traits before and after bariatric surgery, with larger sample sizes and a longer follow-up duration.

### Strength and limits

This study presents several limitations that should be considered. First of all, the small sample size at final follow-up assessment could have led to possible bias in the statistical analyses. Secondly, the use of self-report questionnaires could imply reliance issues, as well as the retrospective assessment of some clinical variables such as previous hypomanic episodes, history of neurodevelopmental disorders, etc. In addition, the psychiatric evaluation in this study was part of the pre-surgical assessment, and this may have led subjects to underestimate some aspects of their psychiatric status or history in order to access surgery. Finally, we were only able to provide data regarding lifetime psychiatric comorbidities, without indicating the prevalence of current disorders. On the other hand, the major strength of this study is the assessment not only of major psychiatric disorders but also of psychopathological traits that may underlie some maladaptive eating behaviors significantly affecting weight outcome. Moreover, this study is based on psychiatric assessments conducted by experienced physicians through the use of structured diagnostic interviews.

### What is already known on this subject?

Several psychiatric disorders are thought to have an impact on the weight loss outcome after bariatric surgery, both in the short- and the long-term. Furthermore, recent studies have suggested that some trans-nosographic psychopathological traits could be predictors of weight loss program success and bariatric surgery outcomes.

### What this study adds?

The present study confirms the association between binge eating disorder, especially when in comorbidity with mood disorders, and a worse outcome in terms of weight loss 1 year after bariatric surgery. Moreover, it is one of the first studies to highlight the important role that emotional dysregulation probably plays in weight control and eating behaviors.

## Data Availability

The datasets generated analyzed during the current study are available from the corresponding author on reasonable request.
